# Supplementary material for: Time trends in mental health indicators in Germany's adult population before and during the COVID-19 pandemic
Source: Front Public Health. 2023 Feb 23;11:1065938. doi: 10.3389/fpubh.2023.1065938 (PMC9995751; doi:10.3389/fpubh.2023.1065938)
Supplement: Supplementary file 1 [file Data_Sheet_1.pdf]

APPENDIX

Time trends in mental health indicators in Germany's adult population before and during the COVID-19 pandemic

Elvira Mauz[1*†], Lena Walther[1†], Stephan Junker[1], Christina Kersjes[1], Stefan Damerow[1], Sophie Eicher[1], Heike Hölling[1], Stephan Müters[1], Diana Peitz[1], Susanne Schnitzer[2], Julia Thom[1]

1 Department of Epidemiology and Health Monitoring, Robert Koch Institute, Berlin, Germany

2 Institute of Medical Sociology and Rehabilitation Sciences, Charité-Universitätsmedizin, Berlin, Germany

*Correspondence:

Elvira Mauz

MauzE@rki.de

Figure A1: Observations per one-month period ranging from middle of the month to middle of the following month

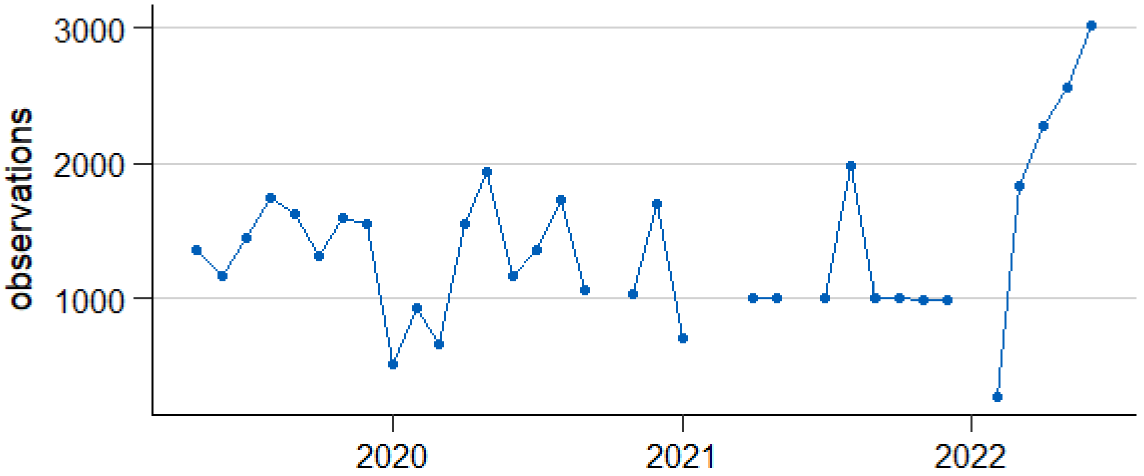

Table A1: p-values for joint tests for changes in scores over time in the population and subgroups by sex, age, and level of education, as well as for changes in differences between subgroups over time

	**cw 11-37 / cw 11-24**		**cw 38-52**	
	changing estimates over time	changing differences over time	changing estimates over time	changing differences over time
Total				
PHQ-2 mean	0.000		0.016	
PHQ-2 > 2	0.000		0.158	
GAD-2 mean	0.000[1]		-	
GAD-2 > 2	0.000[1]		-	
SRMH mean	0.001[1]		-	
SRMH > 3	0.000[1]		-	
Sex				
PHQ-2 mean	0.000	0.400	0.066	0.447
PHQ-2 > 2	0.000	0.298	0.535	0.973
GAD-2 mean	0.000	0.592[1]	-	-
GAD-2 > 2	0.000	0.952[1]	-	-
SRMH mean	0.002	0.437[1]	-	-
SRMH > 3	0.001	0.737[1]	-	-
Age				
PHQ-2 mean	0.000	0.155	0.080	0.262
PHQ-2 > 2	0.000	0.054	0.045	0.074
GAD-2 mean	0.000	0.034	-	-
GAD-2 > 2	0.000	0.236	-	-
SRMH mean	0.000	0.370	-	-
SRMH > 3	0.000	0.205	-	-
Level of education				
PHQ-2 mean	0.000	0.662	0.108	0.977
PHQ-2 > 2	0.000	0.781	0.108	0.338
GAD-2 mean	0.000	0.598	-	-
GAD-2 > 2	0.000	0.820	-	-
SRMH mean	0.003	0.443	-	-
SRMH > 3	0.000	0.281	-	-

[1] In these cases, p-values correspond to p-values of pairwise comparisons because only two estimates are being compared.

Figure A2: Time trends in depressive symptoms (PHQ-2) by age groups

A2 (A): Mean Score

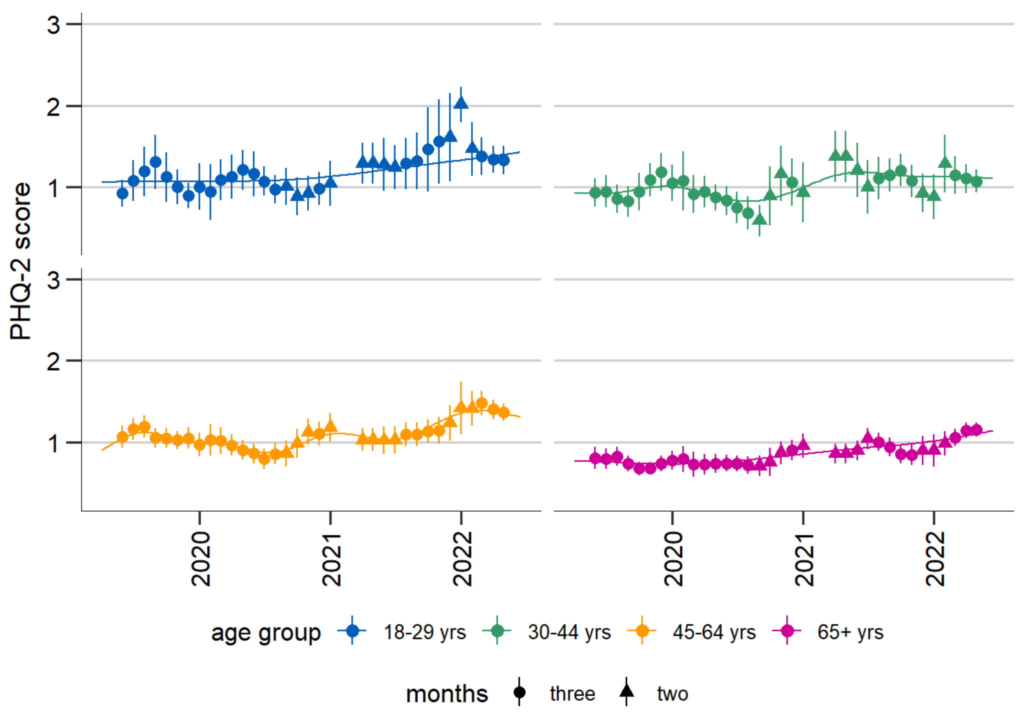

A2 (B): Proportion screening positive

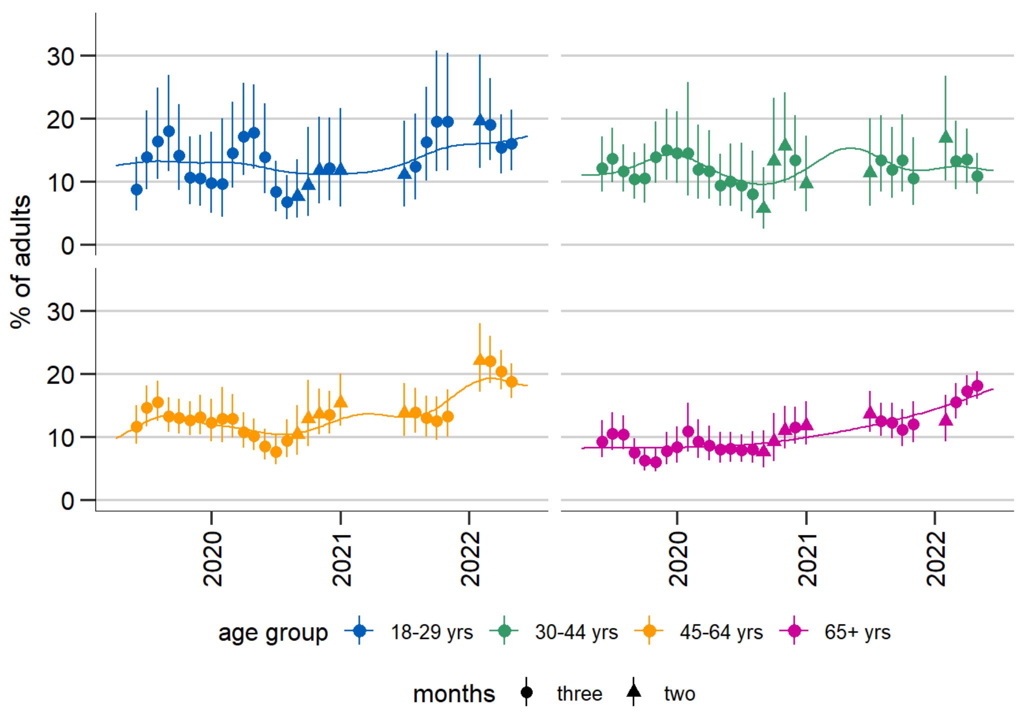

Figure A3: Time trends in anxiety symptoms (GAD-2), total population and by subgroups

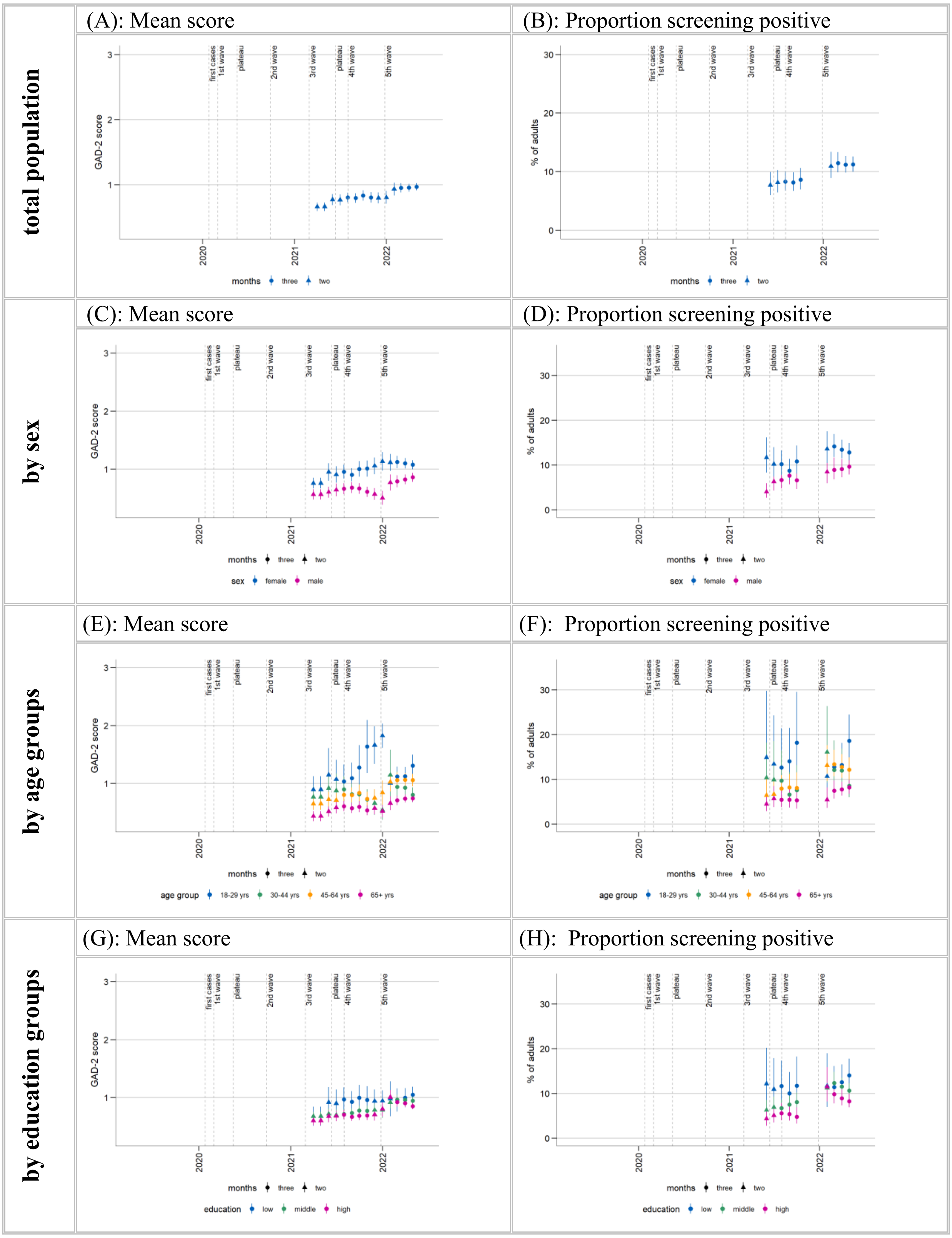

Time series starting from estimate centered on March/April 2021 and ending on estimate centered on April/May 2022. Calculation of three-month moving estimates detailed in section 3.4.1 in Methods. Estimates for each sociodemographic characteristic subgroup (A3 C-H) are standardized for the respective other two characteristics (e.g. estimates for women standardized for age and level of education). Gaps in the time series are due to data gaps. Larger gaps (including at the start of the time series) arise in the time series for proportion of positive screens (A3 B, D, F, H) due to empty cells (absence of positive screens within certain sex, age, and level of education interaction cells in the regression model).

Figure A4: Time trends in anxiety symptoms (GAD-2) by age groups

(A): Mean Score

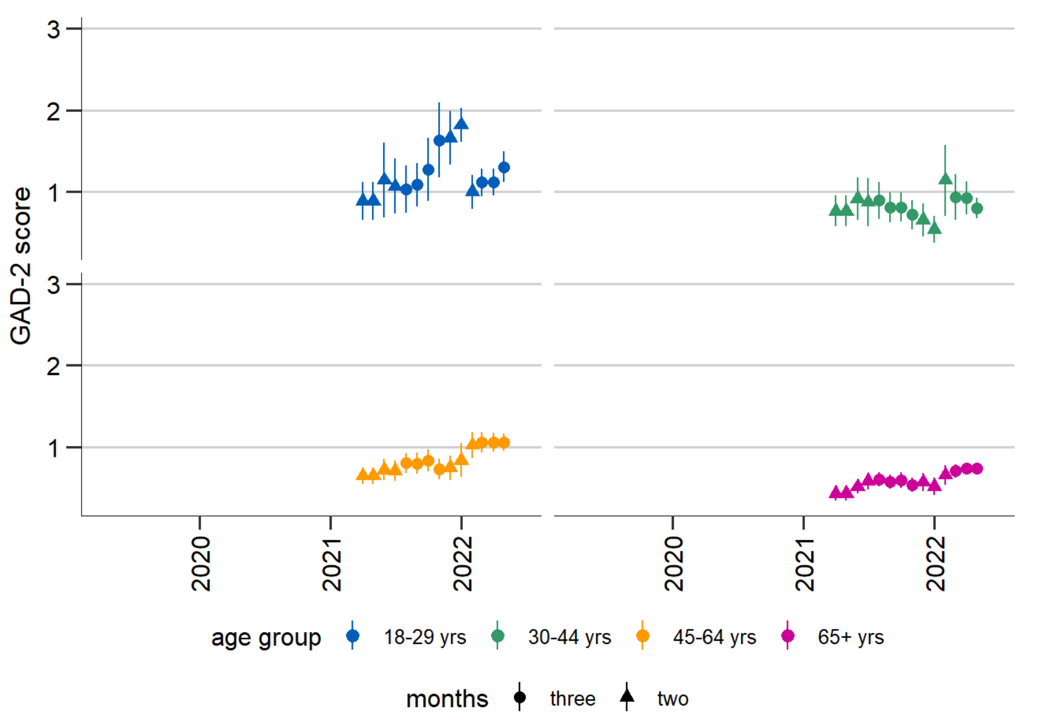

(B): Proportion screening positive

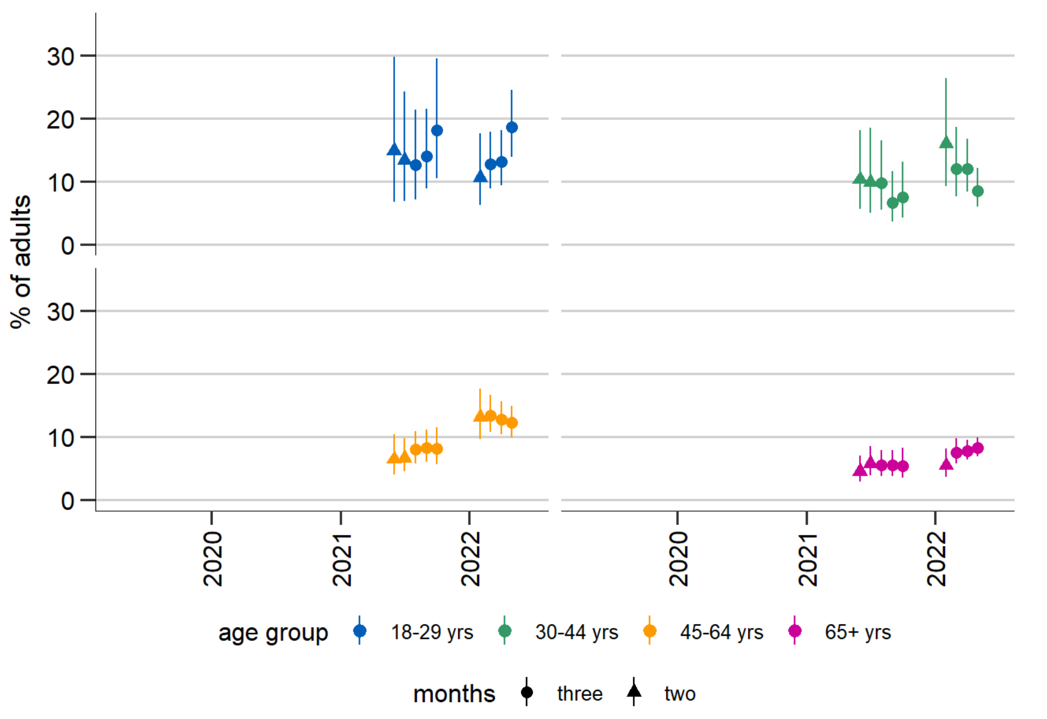

Table A2 (A): Estimated mean anxiety symptom scores (GAD-2) for different time periods and p-values for statistical comparison

	cw 11-37 2021			cw 38-52 2021			cw 11-24 2022			p-value cw 11-37 2021 vs. cw 11-24 2022
	mean	95%-CI		mean	95%-CI		mean	95%-CI		
**Total**	0.75	0.70	0.81	0.81	0.72	0.89	0.96	0.91	1.02	0.000
**Sex**										
male	0.62	0.55	0.69	0.61	0.51	0.71	0.85	0.78	0.93	0.000
female	0.89	0.80	0.97	1.02	0.88	1.16	1.08	1.00	1.15	0.001
**Age**										
18-29	0.99	0.77	1.20	1.68	1.20	2.15	1.30	1.12	1.49	0.029
30-44	0.84	0.68	1.00	0.70	0.52	0.87	0.80	0.68	0.92	0.689
45-64	0.74	0.65	0.83	0.75	0.62	0.88	1.05	0.95	1.16	0.000
65+	0.54	0.48	0.61	0.55	0.46	0.64	0.74	0.68	0.80	0.000
**Level of education**										
low	0.86	0.71	1.01	0.96	0.71	1.20	1.03	0.90	1.17	0.093
middle	0.70	0.64	0.75	0.79	0.69	0.88	0.95	0.88	1.01	0.000
high	0.66	0.61	0.72	0.71	0.62	0.80	0.86	0.80	0.91	0.000

Table A2 (B): Estimated percentages of positive screens for possible anxiety disorder (GAD-2 score > 2) for different time periods and p-values for statistical comparison

	cw 11-37 2021			cw 38-52 2021			cw 11-24 2022			p-value cw 11-37 2021 vs. cw 11-24 2022
	%	95%-CI		%	95%-CI		%	95%-CI		
**Total**	7.2	6.2	8.5				11.1	9.9	12.5	0.000
**Sex**					*					
male	5.6	4.3	7.3				9.5	7.8	11.5	0.001
female	9.0	7.2	11.1				12.8	11.0	14.8	0.005
**Age**										
18-29	10.2	6.1	16.6				18.5	13.8	24.2	0.027
30-44	8.0	5.1	12.2				8.5	6.0	11.9	0.834
45-64	7.1	5.5	9.1				12.1	9.8	14.8	0.001
65+	4.7	3.4	6.4				8.1	6.8	9.7	0.001
**Level of education**										
low	9.2	6.4	13.0				13.8	10.8	17.5	0.054
middle	6.3	5.2	7.6				10.5	9.1	12.2	0.000
high	4.9	3.8	6.3				8.3	7.0	10.0	0.000

Calculation of estimates as well as p-values for comparisons between time periods detailed in section 3.4.2 in Methods. Estimates for each sociodemographic characteristic subgroup are standardized for the respective other two characteristics (e.g. estimates for women standardized for age and level of education). p-values for pairwise comparisons only reported in case of significant joint tests (Table A1). Estimates for percentages of positive screens for CW38-52 could not be calculated due to empty cells (absence of positive screens within certain sex, age, and level of education interaction cells in the regression model).

Figure A5: Time trends in self-rated mental health; total population and by subgroups

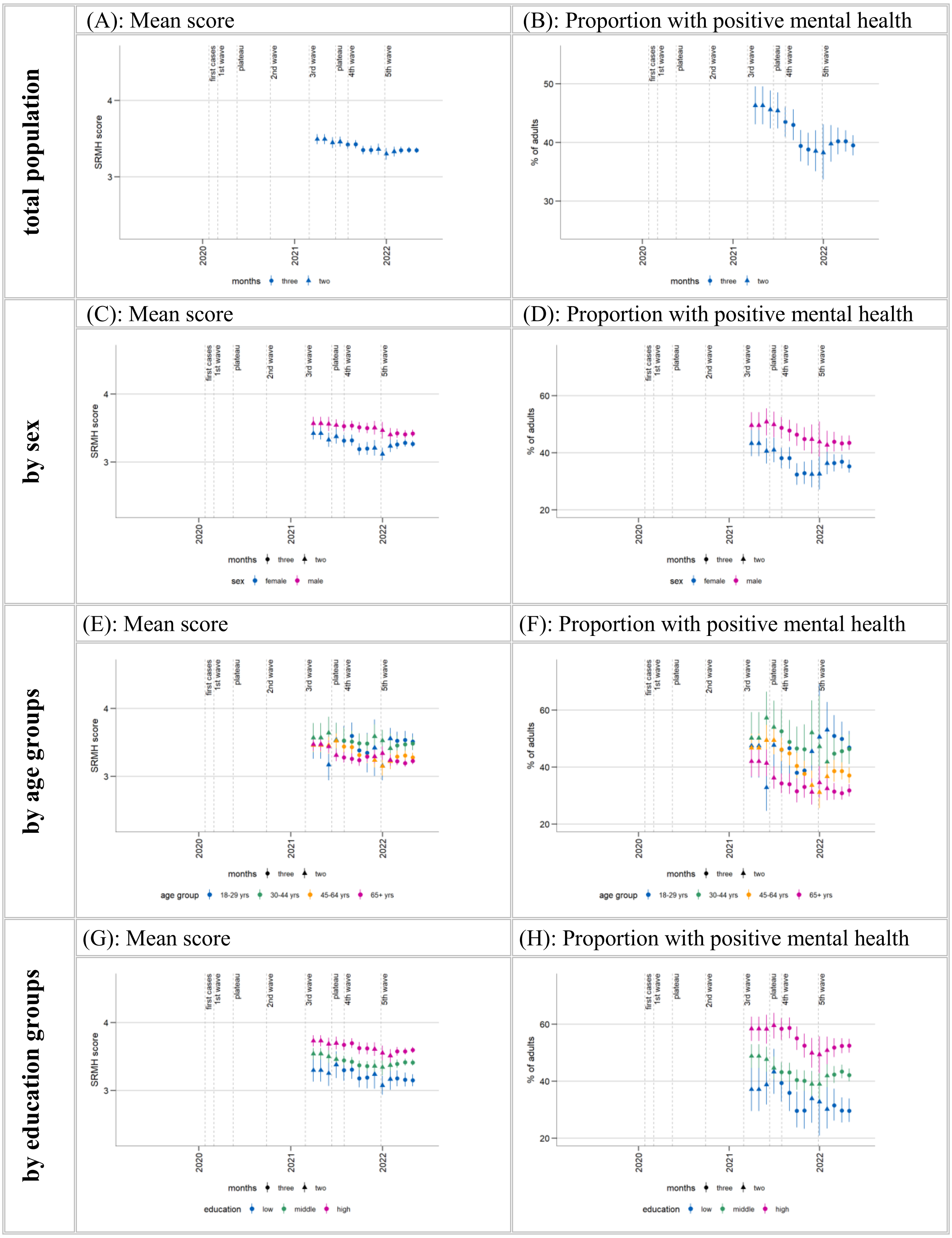

Time series starting from estimate centered on March/April 2021 and ending on estimate centered on April/May 2022. “Proportion with positive mental health” is the proportion who rated their mental health as “very good” or “excellent”. Calculation of three-month moving estimates detailed in section 3.4.1 in Methods. Estimates for each sociodemographic characteristic subgroup (A5 C-H) are standardized for the respective other two characteristics (e.g. estimates for women standardized for age and level of education).

Figure A6: Time trends in self-rated mental health by age groups

(A): Mean Score

(B): Proportion with positive mental health

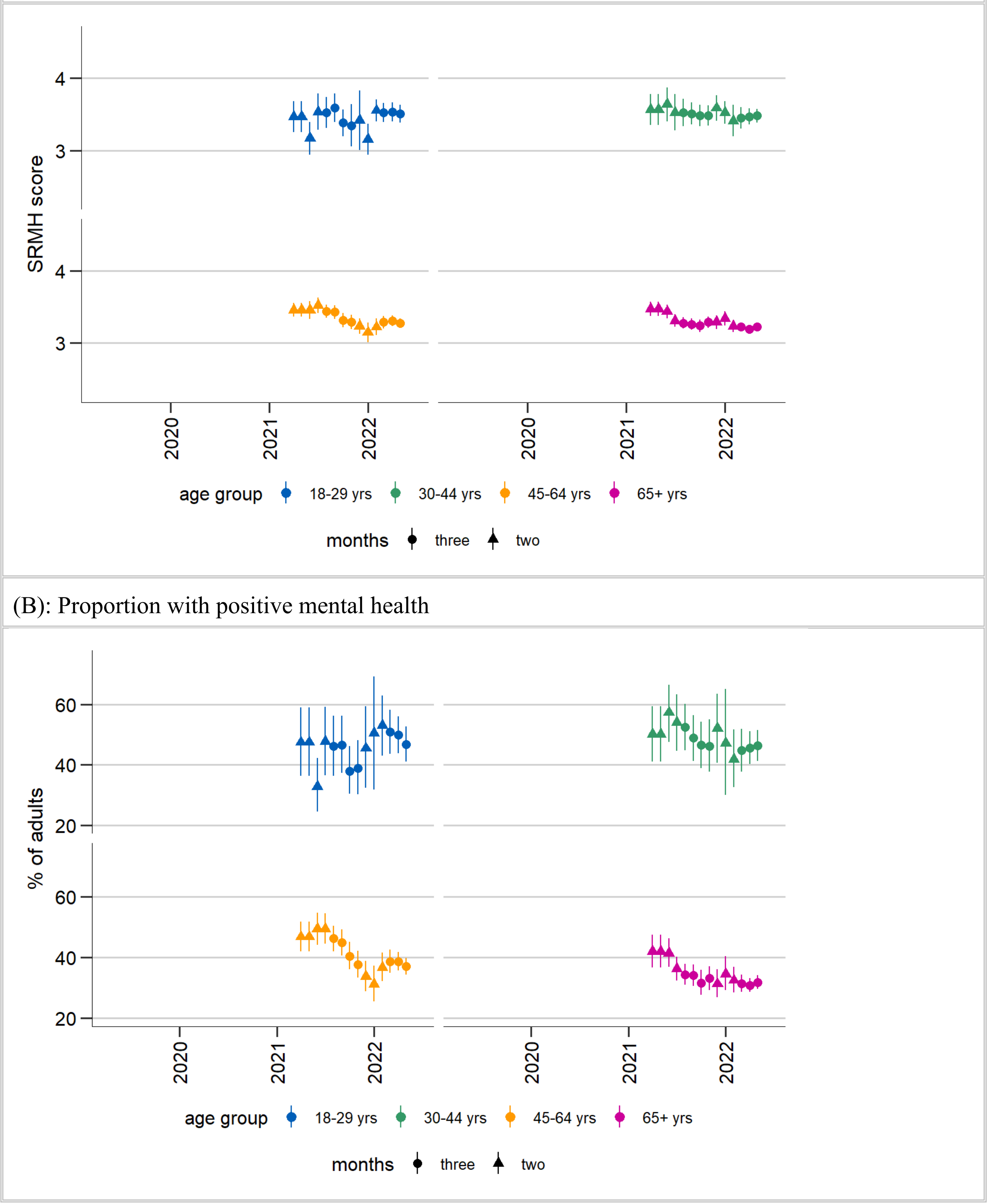

Table A3 (A): Estimated mean SRMH for different time period and p-values for statistical comparisons

	cw 11-37 2021			cw 38-52 2021			cw 11-24 2022			p-value
	mean	95%-CI		mean	95%-CI		mean	95%-CI		cw 11-37 2021 vs. cw 11-24 2022
**Total**	3.44	3.40	3.48	3.35	3.29	3.41	3.35	3.31	3.38	0.001
**Sex**										
male	3.54	3.48	3.60	3.50	3.41	3.58	3.42	3.37	3.48	0.003
female	3.34	3.27	3.40	3.20	3.10	3.29	3.27	3.22	3.31	0.072
**Age**										
18-29	3.50	3.33	3.67	3.38	3.08	3.67	3.51	3.39	3.63	0.900
30-44	3.53	3.40	3.67	3.50	3.32	3.67	3.49	3.40	3.58	0.615
45-64	3.44	3.37	3.51	3.27	3.18	3.37	3.28	3.21	3.35	0.001
65+	3.34	3.28	3.40	3.28	3.20	3.36	3.22	3.18	3.27	0.001
**Level of education**										
low	3.30	3.18	3.41	3.19	3.02	3.36	3.16	3.07	3.24	0.061
middle	3.46	3.41	3.52	3.36	3.28	3.44	3.41	3.36	3.46	0.124
high	3.70	3.64	3.75	3.60	3.51	3.69	3.59	3.55	3.63	0.004

Table A3 (B): Estimated percentages of very good/excellent SRMH for different time periods and p-values for statistical comparisons

	cw 11-37 2021			cw 38-52 2021			cw 11-24 2022			p-value
	%	95%-CI		%	95%-CI		%	95%-CI		cw 11-37 2021 vs. cw 11-24 2022
**Total**	44.3	42.3	46.3	38.4	35.4	41.5	39.6	37.9	41.3	0.000
**Sex**										
male	48.9	46.0	51.7	44.7	40.2	49.3	43.6	41.1	46.1	0.007
female	39.7	36.9	42.5	32.2	28.3	36.5	35.3	33.1	37.6	0.017
**Age**										
18-29	45.7	37.8	53.8	40.4	30.7	50.9	47.3	41.6	53.0	0.758
30-44	51.3	45.6	56.9	46.8	36.8	57.1	46.7	41.6	51.7	0.237
45-64	46.3	43.1	49.4	36.6	32.2	41.3	37.1	34.5	39.9	0.000
65+	37.0	34.2	39.9	32.0	28.0	36.2	31.6	29.5	33.7	0.003
**Level of education**										
low	38.0	32.9	43.3	30.8	23.6	39.2	29.9	26.0	34.1	0.017
middle	45.1	42.5	47.7	39.2	35.4	43.1	42.2	39.9	44.5	0.097
high	58.4	55.5	61.2	51.2	46.7	55.7	52.4	50.0	54.8	0.002

Calculation of estimates as well as p-values for comparisons between time periods detailed in section 3.4.2 in Methods. Estimates for each sociodemographic characteristic subgroup are standardized for the respective other two characteristics (e.g. estimates for women standardized for age and level of education). p-values for pairwise comparisons only reported in case of significant joint tests (Table A1).
